# Daratumumab Monotherapy in Severe Patients with AL Amyloidosis and Biopsy-Proven Renal Involvement: A Real Life Experience

**DOI:** 10.3390/jcm9103232

**Published:** 2020-10-09

**Authors:** Dario Roccatello, Roberta Fenoglio, Carla Naretto, Simone Baldovino, Savino Sciascia, Michela Ferro, Daniela Rossi

**Affiliations:** CMID-Nephrology and Dialysis Unit (ERK-net Member)-Center of Research of Nephrology, Rheumatology, and Rare Diseases, Interregional Coordinating Center of the Network of Rare Diseases, G. Bosco Hospital and University of Turin, 10152 Turin, Italy; roberta.fenoglio@unito.it (R.F.); carla.naretto@unito.it (C.N.); simone.baldovino@unito.it (S.B.); savino.sciascia@unito.it (S.S.); ferromi@libero.it (M.F.); daniela.rossi@unito.it (D.R.)

**Keywords:** amyloidosis, daratumumab, light chain

## Abstract

Objectives: This paper aims to describe the clinical experience with Daratumumab (DARA), a first-in-class anti-CD38 human monoclonal IgG1κ antibody monotherapy, in severe patients with AL and biopsy-proven renal involvement. Immunoglobulin light chain (AL) amyloidosis with multi-organ involvement is characterized by short survival. Novel powerful drugs are expanding the therapeutic options. Current treatment of AL amyloidosis, which has been adopted from multiple myeloma (MM), is based on chemotherapy targeting the underlying plasma cell clone. DARA is effective in treating MM. The clinical activity and toxicity profile of DARA as a single agent in the treatment of AL amyloidosis is currently under evaluation. Patients and Methods: DARA was administered in a series of patients with severe AL amyloidosis and biopsy-proven renal involvement. Five patients(mean age 64.2 years) were treated. One patient was refractory and one intolerant to conventional bortezomib-based therapy, two were treated with DARA for relapsing disease, and one was treated front-line. Results: Data showed that DARA monotherapy resulted in good clinical results, with the disappearance of M-proteins in four out of five patients and with serum free light chains (sFLC) ratio normalization in three out of four and a remarkable amelioration in the remaining patient. The four patients with still preserved renal function at baseline also showed serum creatinine stabilization or improvement and a decrease in proteinuria. These data were paralleled by the reduction of the N-terminal prohormone of brain natriuretic peptide (NT pro-BNP)values. Conclusions: Our data show that monotherapy with DARA had significant clinical efficacy in pretreated/naïve patients with severe AL amyloidosis and biopsy-proven renal involvement.

## 1. Introduction

Immunoglobulin light chain (AL) amyloidosis with multi-organ involvement is characterized by short survival. Outcomes are especially poor for patients who have cardiac and/or renal involvement. In recent years, our understanding of the pathogenesis of AL amyloidosis and the mechanisms of organ involvement has greatly improved, and a good deal of progress has been made in biomarker-based risk stratification and disease monitoring. As a consequence, novel powerful drugs are expanding the therapeutic options [1]. Overall, these advances are resulting in improved outcomes [2]. Traditional treatment of AL amyloidosis includes the use of stem cell transplantation in a minority of patients, while the majority are treated with proteasome inhibitors, alkylating agents, and/or steroid-based combinations [3]. Indeed, current treatment of AL amyloidosis has been derived from multiple myeloma (MM), and is based on chemotherapy targeting the underlying plasma cell clone. Achieving the disappearance (i.e., complete hematological response) or at least a reduction in the involved free light chains (FLC) is considered a critical endpoint which is thought to be crucial for preventing further organ damage [4]. 

Daratumumab (DARA)is a first-in-class anti-CD38 human IgG1κ monoclonal antibody (MoAb) that is effective in treating relapsed/refractory multiple myeloma both as monotherapy and in combination with proteasome inhibitors and immunomodulatory drugs [5,6].The clonal plasma cells in AL amyloidosis express CD38, thus making DARA a potentially effective treatment [7]. Early data from phase I and phase II studies show that DARA is well tolerated in this population of patients and that it induces rapid and in-depth response. The clinical activity and toxicity profile of DARA as a single agent in the treatment of AL amyloidosis is currently under evaluation (NCT04131309). We describe a specific experience with DARA monotherapy in a series of patients with severe AL and biopsy-proven renal involvement.

## 2. Patient Cohort

Five patients (two males and three females), mean age 64.2 years (range 52–69), affected by AL amyloidosis with biopsy-proven renal involvement, were treated with DARA following antibody testing and extended red blood cell (RBC) antigen phenotyping. The treatment protocol included 16 mg/kg DARA administered intravenously weekly for 8 consecutive weeks, then every two weeks eight more times, and lastly monthly until the 52nd week. Premedication included paracetamol 1000 mg (oral), chlorphenamine 10 mg (intravenous), methylprednisolone 125 mg (intravenous). 

Demographics, clinical features, and response to therapy are reported in Table 1 and Table 2. Table 3 includes histological information from kidney biopsy. 

**Case 1** (a bortezomib-based regimen resistant case). The patients had a history of hypertension and presented with nephrotic syndrome to our division. Assessment of M component showed IgAλ, with FLC λ 670 mg/L, FLC ratio 0.02. Renal biopsy confirmed AL amyloidosis, and bone marrow showed a λ-restricted plasma cell clone (7%) positive with Congo red staining. Clinical, laboratory, and radiological tests revealed class I cardiac failure according to the New York Heart Association (NYHA) classification and a peripheral nervous system involvement. The patient was treated with five courses of bortezomib (1.3 mg/m^2^), cyclophosphamide (1000 mg/m^2^), and dexamethasone (160 mg/week) (VCD), without response. He was subsequently treated with four courses of melphalan (10 mg/day) and dexamethasone (20 mg/day) for 4 days/month, but further progressed with an estimated glomerular filtration rate (eGFR) dropping from 70 to 15 mL/min/1.73 m^2^, unchanged proteinuria (12 g/24 h), and an increase in N-terminal prohormone of brain natriuretic peptide (NT pro-BNP)from 593 to 8296 pg/mL.

He underwent a second renal biopsy which showed an increase in amyloid deposits and a bone marrow biopsy which revealed an unchanged percentage of plasma cells. Treatment with DARA resulted in a decrease in NT pro-BNP levels (from 8296 to 4526 pg/mL), disappearance of serum M-component, and Bence–Jones proteinuria and normalization of FLC ratio. However, because of a persistently impaired renal function with eGFR of 7 mL/min/1.73 m^2^, hemodialysis was started. No infusion-related reactions or adverse events were observed with DARA. Treatment was discontinued at the 52nd week and a third renal biopsy was performed. The amyloid deposits were unchanged, but there was a significant increase in sclerotic glomeruli and interstitial fibrosis. The patient is currently being evaluated for kidney transplantation.

**Case 2** (a relapsing case). The patient presented with anemia (hemoglobin 10.7 g/dL), nephrotic syndrome (proteinuria 4.5 g/24 h), asthenia, and paresthesia of the lower limbs. Assessment of M component was positive for light chain λ. Renal biopsy showed AL amyloidosis, and bone marrow analysis revealed a λ-restricted plasma cell clone (8%). Clinical, laboratory, and radiological tests indicated cardiac and peripheral nervous system involvement. The patient was treated with nine courses of melphalan i.v. and dexamethasone (20 mg/day) for 4 days/month, which resulted in a complete response. Two years later, the FLC ratio decreased to 0.22, with FLC λ 73.5 mg/L, and proteinuria increased from 1.2 to 6.8 g/24 h with reappearance of Bence–Jones proteinuria. This was paralleled by a relapse of lower limb paresthesia. Renal biopsy confirmed an increase in amyloid deposits, electromyography revealed a severe peripheral neuropathy, and bone marrow analysis showed a λ-restricted plasma cell clone 7%. Treatment with DARA resulted in a rapid decrease in proteinuria (from 6.8 to 2.7 g/24 h at the 16th dose) and NT pro-BNP levels (from 1844 to 330 pg/mL) with disappearance of serum M component and Bence–Jones proteinuria and normalization of FLC ratio. Again, no infusion-related reactions or adverse events were observed. Treatment is currently ongoing.

**Case 3** (a front-line administration case). The patient had a previous a history of intestinal diverticulosis (for which he underwent surgical resection in 2008) and presented with nephrotic syndrome (proteinuria 9.3 g/24 h), Bence–Jones proteinuria, and renal failure (sCr 2.4 mg/dL). Assessment of M component was positive for light chain λ with FLC λ 390 mg/L and FLC ratio 0.06. Renal biopsy showed AL amyloidosis, and bone marrow analysis revealed a λ-restricted plasma cell clone (9% of infiltration). Clinical, laboratory, and radiological tests indicated class I NYHA-, New York Heart Association cardiac failure. Treatment with DARA resulted in a rapid decrease in proteinuria (from 9.3 to 2.2 g/24 h at the 16th dose) with weakly positive Bence–Jones proteinuria, normalization of FLC ratio and disappearance of serum M component, and reduction in NT pro-BNP levels from 850 to 225 pg/mL. No infusion-related reactions or adverse events were noted. Treatment is currently ongoing.

**Case 4** (a bortezomib-based regimen intolerant case).The patient had a history of severe malabsorption syndrome, for which parenteral nutrition was started two years before. The patient presented with cachexia and depressive syndrome. Laboratory tests revealed nephrotic syndrome (proteinuria 8.2 g/24 h), Bence–Jones proteinuria associated with marked hypoprotidemia (serum albumin 1.2 g/dL and total serum proteins of 3.5 g/dL). She had an IgDλ M component with FLC λ 134 mg/L and FLC ratio 0.12. Tissue histological examination confirmed the clinical suspicion of amyloid deposits in the kidney, intestinal mucosa, and periombelical fat. Bone marrow showed a λ-restricted plasma cell clone (13%), and testing with Congo red staining was positive. No signs of cardiac or peripheral nervous system involvement were found. The patient was initially treated with bortezomib (1.3 mg/m^2^), cyclophosphamide (1000 mg/m^2^), and dexamethasone (160 mg/week), but the protocol was discontinued after the first cycle because of intolerance and the appearance of leukocytopenia. Treatment with DARA resulted in a rapid decrease in proteinuria (from 8.2 to 1.9 g/24 h at the 13th dose) with disappearance of Bence–Jones proteinuria and serum M component and normalization of FLC ratio. No infusion related reactions were observed. After the 13thinfusion, the patient was hospitalized for pneumonia and treatment was temporarily discontinued.

**Case 5** (a relapsing case). The patient had a history of hypothyroidism and presented with nephrotic syndrome (proteinuria 4.7 g/24 h). Assessment of M component was positive for IgGλ. Renal biopsy showed AL amyloidosis, and bone marrow analysis revealed a λ-restricted plasma cell clone (12%). Clinical and electrophysiologic tests indicated peripheral nervous system involvement, while cardiac involvement was excluded. The patient was treated with nine courses of Lenalidomide (15 mg/day, day 1–21), cyclophosphamide (300 mg/m^2^, days 1-8-15), and dexamethasone (160 mg/month), which resulted in a complete response. Six years later, the FLC ratio decreased to 0.21, FLC λ was 73.5 mg/L and total proteinuria and Bence Jones proteinuria reappeared (2.5 g/24 h). Renal biopsy showed amyloid deposits. Bone marrow analysis revealed a λ-restricted plasma cell clone (7%). No cardiac or peripheral nervous system involvement was detected. Treatment with DARA resulted in a rapid decrease in proteinuria (from 2.5 to 1 g/24 h at the 13th dose) and serum M component and an increase in FLC ratio (0.29, nv: 0.31–1.56), with weakly positive Bence–Jones proteinuria. The patient experienced an infusion reaction during the first dose (grade 1). No other adverse events were noted. Treatment is currently ongoing.

## 3. Discussion

AL amyloidosis is a rare disease caused by clonally restricted plasma cells that produce monoclonal light chains which form insoluble fibrils that are then deposited into vital organs including the heart in 60% of cases, and the kidney in 70%, thus inducing irreversible cell damage [8].

Survival of patients with AL amyloidosis is widely heterogeneous and depends on the degree of organ involvement. Advanced cardiac involvement is associated with the worst outcome [2]. Kidney involvement is not directly associated with reduced survival but does influence both access to innovative therapy and quality of life [9].

Prognostic classifications include the standard Mayo Staging System (MSS) (which considers cardiac troponins and NT pro-BNP [10]), the 2014 revised MSS version (that incorporates the level of pathologic light chain [11]), and a more recently proposed Staging System using BNP detection (which is more commonly available than NT pro-BNP and is less influenced by renal impairment [12]). A staging system based on glomerular filtration rate (GFR) and proteinuria at diagnosis has been found to predict the risk of progression to end-stage kidney disease in a large cohort of patients with a presumptive diagnosis of kidney amyloidosis (mostly based on clinical grounds) [7]. However, whether this staging system can be applied to patients with biopsy-proven renal involvement is presently speculative.

The goals of therapy in AL amyloidosis is a suppression of the production of the pathologic light chain precursor in order to improve organ impairment. This is difficult to achieve because the process of amyloid deposition, at least in the kidney, is hardly reversible [13]. Indeed, repeat biopsies show the persistence of deposits even in patients with renal response. When treated with bortezomib-based regimens, patients with renal involvement show a complete hematologic response in half of the cases. Hematologic response is an essential prerequisite for maintaining or improving renal function. Indeed, a >90% response in free light chains at 6 months has been associated with a four-fold increase in the likelihood of renal response [14]. A delay in hematologic response results in further deposition of amyloid that reduces the chances of renal improvement. This is why a more aggressive treatment than the conventional bortezomib-based regimen is desirable ab initio in these high-risk patients.

DARA is a highly effective anti-plasma cell therapy that has become integral in the treatment of multiple myeloma. It has rapidly shifted from being an agent to be used in relapsed/refractory settings to a drug with a putative role in the early disease course. Clinical trials are currently investigating its efficacy in the front-line setting [15].

Previous studies have shown encouraging data regarding the safety, tolerability, and effectiveness (with a hematologic response rate >80) of daratumumab-based therapies in AL amyloidosis [16,17,18,19].

Renal response has not been specifically addressed in these studies.

In a phase II study, Sanchorawala et al. also reported high hematologic response rates (>80%) in 21 patients with relapsed AL amyloidosis [20].

In another cohort, 84 patients with previously treated AL amyloidosis who received DARA (with dexamethasone alone or in combination with other plasma-cell directed therapies) were retrospectively evaluated for survival and organ response. Sixty-seven (84%) out of 80 evaluable patients achieved a hematologic response. Sixty-eight patients (81%) had cardiac involvement, and 53% of evaluable patients achieved a cardiac response with a median initial response time of 2 months among responders. Of the 53 patients with renal impairment or urinary abnormalities, 26 were evaluable and achieved some renal response, with a median initial response time of 6 months [21]. However, none of the patients in these series were reported as having biopsy-proven renal involvement.

The presence of amyloids unique proof of kidney involvement. Due to the several causes of proteinuria in the elderly, renal biopsy should be performed in all cases of urinary abnormalities despite the finding of positive Congo red staining in another tissue. The extent and distribution of renal amyloid deposition might also be important when comparing the outcomes of these patients [22].

The results of a prospective multicenter phase II study of DARA monotherapy in AL amyloidosis have been very recently published [23].Forty previously treated patients from 15 centers were examined. They included 26 patients with renal involvement. They had no renal biopsy and no insight into the definition of renal involvement had been given. Twenty-one of them had <60 mL/mi of eGFR. This figure, considering patients’ mean age (69 years), is close to normal. Seven patients were claimed to have a renal response because of a 30% decrease in proteinuria without a 25% increase in eGFR, but no detail of the baseline proteinuria levels had been given.

Taken together, these results are difficult to interpret.

When compared to the available evidence, while confirmation in a larger study is needed, nevertheless, the marked decrease in proteinuria and Bence–Jones protein, along with the disappearance of the M component, represent a major point of interest in our study.

## 4. Conclusions

The present study describes five patients with AL amyloidosis and biopsy-proven renal involvement who underwent DARA monotherapy in a single center. Our data, based on real life experience and strong parameters of definition of the renal involvement, showed this regimen to be a beneficial therapeutic option capable not only of inducing rapid hematologic response but also achieving a substantial rate of renal improvement in pretreated and naïve patients.

## Figures and Tables

**Table 1:** Baseline clinical laboratory data.

	Age at the Start of Therapy	M Component	% BM Infiltration	NYHA Class	Neurologic Involvement	Previous Treatment	FLC Ratio 0.31–1.56	B2 Micro-Globulin	NT Pro-BNP Levels T0 pg/mL	Bence–Jones T0	sCr T0 mg/dL	uPT T0 g/Day
Pt 1	68	IgAλ	7%	II	+	B-CYC-D M-D	0.02	16.8	8296	++	HD	−−
Pt 2	55	λ	8%	I	++	M-D	0.22	1.9	1844	++	0.7	6.8
Pt 3	70	λ	9%	I	−	−−	0.06	3.79	850	++	2.3	9.3
Pt 4	67	IgDλ	13%	−−	−−	B-CYC-D (1 cycle)	0.12	4.53	175	++	0.4	8.2
Pt 5	72	IgGλ	**7%**	−−	−−	L-CYC-D	0.21	2.3	328	++	0.8	2.5

B: bortezomib, CYC: cyclophosphamide, D: dexamethasone, M: melphalan, L: lenalidomide, FLC: free light chain, sCr: creatinine, uPT: proteinuria, BM, bone marrow, NYHA, New York Heart Association.-terminal prohormone of brain natriuretic peptide (NT pro-BNP).

**Table 2:** Clinical laboratory data after therapy.

		M Component	NYHA Class	FLC Ratio 0.31–1.56	Pro-BNP Levels pg/mL (5–125)	Bence–Jones	sCr mg/dL (0.6–1.2)	uPT g/Day	Adverse Effects
Pt 1	13th dose	A	I	0.49	9048	−−	−−	−−	0
	End FU	A	I	0.53	4526	−−	−−	−−	0
Pt 2	13th dose	A	I	1.06	337	Neg	0.7	2.8	0
	End FU	A	I	1.05	330	Neg	0.8	2.7	0
Pt 3	13th dose	R	I	0.1	213	+/−	1.7	2.3	0
	End FU	A	I	0.25	225	Neg	1.5	2.2	0
Pt 4 *	13th dose	A	−−	0.77	177	Neg	0.5	1.9	Pneumonia
	End FU								
Pt 5 *	13th dose	R	−−	0.29	369	+	0.7	1	0
	End FU								

FLC: free light chain, sCr: creatinine, uPT: proteinuria, A:absent, R: reduced, FU: follow-up. * For pt 4 and pt 5, the 13th dose match.

**Table 3:** Distribution of amyloid deposits in the kidney (biopsy performed before starting therapy).

	Glomerular Involvement	Interstitial Involvement	Vascular Involvement	IF
Pt 1	38/38	+	++	Lambda +++
Pt 2	31/34	+	++	Lambda: +++
Pt 3	17/17	+++	++	Lambda: +++
Pt 4	14/18	++	+++	Lambda: +++
Pt 5	18/24	+	+/−	Lambda +++

IF, immunofluorescence.

## References

[1] Palladini G., Merlini G. (2016). What is new in diagnosis and management of light chain amyloidosis?. Blood.

[2] Muchtar E., Gertz M.A., Kumar S.K., Lacy M.Q., Dingli D., Buadi F.K., Grogan M., Hayman S.R., Kapoor P., Leung N. (2017). Improved outcomes for newly diagnosed AL amyloidosis between 2000 and 2014: Cracking the glass ceiling of early death. Blood.

[3] Sher T., Fenton B., Akhtar A., Gertz M.A. (2016). First report of safety and efficacy of daratumumab in 2 cases of advanced immunoglobulin light chain amyloidosis. Blood.

[4] Palladini G., Comenzo R.L. (2012). The Challenge of Systemic Immunoglobulin Light-Chain Amyloidosis (AL). Subcell. Biochem..

[5] Dimopoulos M.A., Oriol A., Nahi H., San-Miguel J., Bahlis N.J., Usmani S.Z., Rabin N., Orlowski R.Z., Komarnicki M., Suzuki K. (2016). Daratumumab, Lenalidomide, and Dexamethasone for Multiple Myeloma. N. Engl. J. Med..

[6] Palumbo A., Chanan-Khan A., Weisel K., Nooka A.K., Masszi T., Beksac M., Spicka I., Hungria V., Munder M., Mateos M.V. (2016). Daratumumab, Bortezomib, and Dexamethasone for Multiple Myeloma. N. Engl. J. Med..

[7] Khouri J., Kin A., Thapa B., Reu F.J., Bumma N., Samaras C.J., Liu H.D., Karam M.A., Reed J., Mathur S. (2018). Daratumumab proves safe and highly effective in AL amyloidosis. Br. J. Haematol..

[8] Merlini G., Dispenzieri A., Sanchorawala V., Schönland S.O., Palladini G., Hawkins P.N., A Gertz M. (2018). Systemic immunoglobulin light chain amyloidosis. Nat. Rev. Dis. Prim..

[9] Varga C., Titus S.E., Toskic D., Comenzo R.L. (2019). Use of novel therapies in the treatment of light chain amyloidosis. Blood Rev..

[10] Dispenzieri A., A Gertz M., Kyle R.A., Lacy M.Q., Burritt M.F., Therneau T.M., Greipp P.R., Witzig T.E., Lust J.A., Rajkumar S.V. (2004). Serum Cardiac Troponins and N-Terminal Pro-Brain Natriuretic Peptide: A Staging System for Primary Systemic Amyloidosis. J. Clin. Oncol..

[11] Palladini G., Milani P., Basset M., Russo F., Lavatelli F., Nuvolone M., Ferraro G., Bozzola M., Foli A., Perlini S. (2017). Severity and reversibility of cardiac dysfunction and residual concentration of amyloidogenic light chain predict overall survival of patients with AL amyloidosis who attain complete response. Amyloid.

[12] Palladini G., Foli A., Milani P., Russo P., Albertini R., Lavatelli F., Obici L., Perlini S., Moratti R., Merlini G. (2012). Best use of cardiac biomarkers in patients with AL amyloidosis and renal failure. Am. J. Hematol..

[13] Angel-Korman A., Jaberi A., Sanchorawala V., Havasi A. (2019). The utility of repeat kidney biopsy in systemic immunoglobulin light chain amyloidosis. Amyloid.

[14] Pinney J.H., Lachmann H.J., Bansi L., Wechalekar A.D., Gilbertson J.A., Rowczenio D., Sattianayagam P.T., Gibbs S.D., Orlandi E., Wassef N.L. (2011). Outcome in Renal AL Amyloidosis After Chemotherapy. J. Clin. Oncol..

[15] Mateos M.-V., Dimopoulos M.A., Cavo M., Suzuki K., Jakubowiak A., Knop S., Doyen C., Lúcio P., Nagy Z., Kaplan P. (2018). Daratumumab plus Bortezomib, Melphalan, and Prednisone for Untreated Myeloma. N. Engl. J. Med..

[16] Sidiqi M.H., Gertz M.A. (2018). Daratumumab for the treatment of AL amyloidosis. Leuk. Lymphoma.

[17] Gran C., Gahrton G., Alici E., Nahi H. (2018). Case Report: Treatment of light-chain amyloidosis with daratumumab monotherapy in two patients. Eur. J. Haematol..

[18] Chakraborty R., Lentzsch S. (2020). Emerging drugs for the treatment of light chain amyloidosis. Expert. Opin. Emerg. Drugs..

[19] Kimmich C., Schönland S., Ziehl R., Ho A.D., Dittrich T., Müller-Tidow C., Hegenbart U. (2017). Daratumumab Monotherapy in Thirty-Two Heavily. Pre-Treated Patients with Advanced Systemic Light-Chain Amyloidosis. Blood.

[20] Sanchorawala V., Sarosiek S., Sloan J.M., Brauneis D., Migre M.E., Mistark M., Santos S., Cruz R., Fennessey S., Shelton A.C. (2018). Safety, Tolerability and Response Rates of Daratumumab in Patients with Relapsed Light Chain (AL) Amyloidosis: Results of a Phase II Study. Blood.

[21] Chung A., Kaufman G.P., Sidana S., Eckhert E., Schrier S.L., Lafayette R.A. (2019). Extended Follow-up and Organ Responses with Daratumumab Therapy in PreviouslyTreated AL Amyloidosis. Blood Adv..

[22] Murakami Y., Hattori S., Sugiyama F., Yoshikawa K., Sugiura T., Matsushima H. (2014). A case of primary (AL) amyloidosis with predominantly vascular amyloid deposition in the kidney. CEN Case Rep..

[23] Roussel M., Merlini G., Chevret S., Arnulf B., Stoppa A.M., Perrot A., Palladini G., Karlin L., Royer B., Huart A. (2020). A prospective phase II of daratumumab in previously treated systemic light chain amyloidosis patients. Blood.

